# Effect of aluminum chloride hemostatic agent on microleakage of class V composite resin restorations bonded with all-in-one adhesive

**DOI:** 10.4317/medoral.17683

**Published:** 2012-05-01

**Authors:** Narmin Mohammadi, Soodabeh Kimyai, Mahmood Bahari, Fatemeh Pournaghi-Azar, Aysan Mozafari

**Affiliations:** 1 DDS, MS: Associate Professor. Department of Operative Dentistry, School of Dentistry, Tabriz University of Medical Sciences, Tabriz, Iran; 2 DDS, MS: Assistant Professor. Department of Operative Dentistry, School of Dentistry, Tabriz University of Medical Sciences, Tabriz, Iran; 3 DDS. Department of Operative Dentistry, School of Dentistry, Tabriz University of Medical Sciences, Tabriz, Iran

## Abstract

Objectives: Since hemostatic agents can induce changes on enamel and dentin surfaces and influence composite resin adhesion, the aim of the present study was to evaluate the effect of the aluminum chloride hemostatic agent on the gingival margin microleakage of class V (Cl V) composite resin restorations bonded with all-in-one adhesive. Study design: Cl V cavities were prepared on the buccal surfaces of 60 sound bovine permanent incisors. Gingival margins of the cavities were placed 1.5 mm apical to the cemento-enamel junction (CEJ). The teeth were randomly divided into two groups of 30. In group 1, the cavities were restored without the application of a hemostatic agent; in group 2, the cavities were restored after the application of the hemostatic agent. In both groups all-in-one adhesive and Z250 composite resin were used to restore the cavities with the incremental technique. After finishing and polishing, the samples underwent a thermocycling procedure, followed by immersion in 2% basic fuschin solution for 24 hours. The samples were sectioned and gingival microleakage was evaluated under a stereomicroscope. The non-parametric Mann-Whitney U test was used to compare microleakage between the two groups. Statistical significance was defined at P<0.05. Results: A statistically significant difference was observed in microleakage between the two groups (P<0.001). Conclusions: Contamination of Cl V composite resin restorations bonded with all-in-one adhesive with aluminum chloride hemostatic agent significantly increases restoration gingival margin microleakage.

** Key words:**All-in-one adhesive resin, composite resin restoration, hemostatic agent, microleakage.

## Introduction

Dentinal seal of restorations is very important and lack of it is referred to as microleakage, which is the main etiologic agent for recurrent caries, tooth hypersensitivity and pulpal irritation subsequent to restorative procedures and the final failure of restoration (1). Adhesion of composite resins to dentin is a highly technique-sensitive and variable process, which is influenced by surface contamination. Composite resins require a clean and contamination-free environment to achieve maximum bond strength. The quality of adhesion of composite resins to dentin depends on a proper interaction between the resin and mineral-free dentin collagen fibers (2); contaminants such as blood and gingival crevicular fluid occlude the dentinal tubules and prevent the resin tags from penetrating into the denuded collagen fibers, thereby interfering with the bonding process and decreasing bond strength (2,3).

One of the methods to control hemorrhage and the gingival crevicular fluid is to use hemostatic agents such as aluminum chloride. However, such agents have acidic pH values and interfere with the bonding procedure of self-etch adhesive resins to dentin by removing the smear layer (4). A study by O’Keefe et al. showed that hemostatic agents decrease bond strength in self-etch adhesive systems (5). However, in a study by Kimmes et al. the use of hemostatic agents did not influence the shear bond strength of composite resins to dentin with total-etch adhesive systems (2). Recently new all-in-one adhesive systems (7th generation one-step self-etch) have been introduced, which have simplified the bonding process. These systems do not require separate etching, rinsing and drying steps; as a result, the possibility of dentin desiccation is eliminated and the time needed for a restorative procedure decreases (6,7). In addition, these systems are not as technique-sensitive as total-etch adhesives, which is considered an advantage. Therefore, these systems have attracted the attention of clinicians and there is an increasing demand for their use in clinical settings (8).

Hemostatic agents can induce changes in enamel and dentin surfaces, influencing the adhesion of composite resin restorations (2). No previous studies have evaluated the effect of such agents on microleakage of composite resin restorations with the use of all-in-one adhesive resins and the results of other adhesive systems cannot be extended to all-in-one adhesive systems due to differences in pH values and monomeric components (9). Therefore, the present in vitro study was undertaken to evaluate the effect of aluminum chloride hemostatic agent on gingival margin microleakage of Cl V cavities restored with composite resin with the use of all-in-one adhesive system.

## Material and Methods

In this vitro study 60 sound permanent bovine mandibular incisors (10,11) were used. The teeth were free of any abrasions, cracks and structural defects as confirmed by visual examination and evaluated under a stereomicroscope (Nikon, Tokyo, Japan) and used within two months following extraction. The teeth were placed in a 0.5% chloramine-T trihydrate bacteriostatic/bacteiocidal solution for one week and then stored in distilled water in a refrigerator at 4ºC. The storage medium was replaced periodically. The teeth were mounted in a cold-curing acrylic resin and kept in cold water until the resin was completely cured in order to avoid the thermal effects generated by the resin curing process. 24 hour prior to the experimental procedures, the teeth were conditioned in distilled water at (23±2)ºC. Then they were randomly divided into two groups of 30 and treated as follows:

In group 1, Cl V cavities were prepared with a high-speed handpiece using a cylindrical diamond bur (Diatech Dental AG, Swiss Dental Instruments, CH-9435 Heerbrugg) on the buccal surfaces of the teeth, with occlusogingival and mesiodistal dimensions of 3×3 mm and a depth of 2 mm (11). The occlusal wall of the cavities were placed 1.5 mm coronal to the CEJ and the gingival wall was placed 1.5 mm apical to the CEJ (12). All the cavity margins were butt-jointed without any bevels (13). Before tooth preparation the dimension of each cavity was drawn on each tooth using standard templates and the depth of prepared cavity was measured by a probe. During preparation tooth surfaces were kept wet all the times to avoid dehydration. Clearfil tri S Bond (Kuraray Medical INC, Tokyo, Japan) one-step self-etch adhesive was used on cavity walls according to manufacturer’s instructions. Astralis 7 halogen light-curing unit (Ivoclar Vivadent, Schaan, Liechtenstein) was used for light-curing procedures; the light-directing probe had a diameter of 8 mm and directed a light ray at an intensity of 400 mW/cm2 perpendicular to the surface, barely touching the surface, for 20 seconds according to manufacturer’s instructions.

A-3 shade of Z250 composite resin (3M ESPE Dental Products, St. Paul, MN, USA) was used to restore the cavities using the incremental technique (two 1-mm layers). Each layer was cured for 20 seconds. Post-curing was carried out for 60 seconds at an intensity of 700 mW/cm2.

In group 2, all the procedures were the same as those in group 1 with exception that before the application of all-in-one adhesive resin the gingival margins were contaminated with aluminum chloride hemostatic agent (Hemostop, Dentsply, Argentina) using a mini-brush; the margins were rinsed for 30 seconds with water spray after 2 minutes and dried with air spray (4).

All the restorative procedures were carried out by one operator. After restorative procedures, the specimens were finished and polished and placed in distilled water at 37ºC for 24 hours in an incubator (14). To simulate oral cavity conditions the specimens were subjected to a thermocycling procedure at 5±2ºC/55±2ºC, consisting of 500 cycles (15) with a dwell time of 30 seconds and 10 seconds for transfer. Then the samples were dried and covered with two layers of nail varnish up to 1 mm from the cavity margins. In addition, the apex of each tooth was sealed with utility wax. The specimens were then placed in 2% basic fuschin for 24 hours (14). The teeth were subsequently divided in half in a buccolingual direction using diamond disks (Diamont Gmbh, D&Z, Berlin, Germany) and evaluated under a stereomicroscope (Nikon, Tokyo, Japan ) at ×16. Both hemisections of each specimen were evaluated. When the scores were different on both sides, the score indicating more microleakage was used in the evaluation. Dye penetration depth at gingival margins was evaluated based on the following classification:

0= no dye penetration.

I= dye penetration along the gingival wall without the axial wall involvement.

II= dye penetration along the gingival wall with the axial wall involvement.

The non-parametric Mann-Whitney U test was used to compare microleakage between the two groups. Statistical significance was defined at P<0.05.

## Results

 Table 1 presents microleakage scores in the two groups under study. The results demonstrated statistically significant differences in microleakage between the two groups (U=112.50, P<0.001). Group 2 (with hemostatic agent) showed significantly increased gingival margin microleakage compared to group 1 (without hemostatic agent).

**Table 1:**
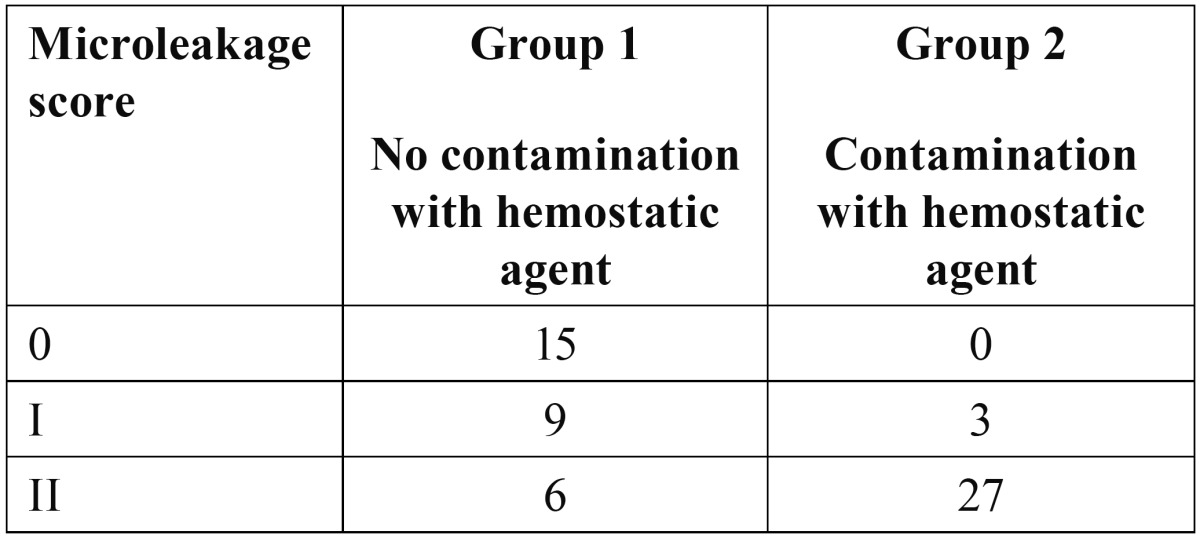
Microleakage scores in the two groups under study.

## Discussion

Composite resins require a clean and contamination-free environment to achieve maximum bond strength (2). Isolation and prevention of surface contamination of prepared tooth surfaces, especially in areas adjacent to gingival tissues, is very difficult (3). Therefore, the present study was undertaken to evaluate the effect of contamination with aluminum chloride hemostatic agent on gingival margin microleakage in Cl V composite resin restorations bonded with all-in-one adhesive resin. The use of aluminum chloride is a common method to control bleeding and gingival crevicular fluid (4).

The results of the present study showed that contamination of the gingival margin with aluminum chloride hemostatic agent significantly increases microleakage at gingival margins of Cl V composite resin cavities, bonded with one-step self-etch adhesive resin. These results are consistent with the results of previous studies indicating that bond strength decreases with the contamination of surface with hemostatic agents in Cl V composite resin restorations, bonded with self-etch adhesive resins (4,5). No studies to date have evaluated the effect of aluminum chloride hemostatic agent on gingival margin microleakage.

The decrease in sealing ability and increase in microleakage subsequent to the use of aluminum chloride hemostatic agent might be attributed to the removal of the smear layer and relative obstruction of the dentinal tubules, which influence the dentin bonding mechanism in the self-etch systems which are dependent on the exposed collagen fibers and modified smear layer (4). In addition, surface contamination with aluminum chloride reduces demineralization after the use of self-etch system, which might be attributed to the fact that the hemostatic agent replaces calcium in hydroxylapatite and produces an insoluble compound. Therefore, demineralization becomes limited in the self-etch systems which have weak acidity. As a result, the bonding mechanism of self-etch systems is influenced (4).

Contrary to the results of the present study, Kuphasuk et al. evaluated the effect of the use of aluminum chloride hemostatic agent on total-etch adhesive systems and did not report any differences in bond strength between contaminated and normal dentin (4). The differences in the results of the two studies might be attributed to differences in the adhesive systems. In the present study, self-etch all-in-one adhesive system was used but in that study total-etch adhesive system was used, which contains phosphoric acid with a high acidity and is capable of simultaneously demineralizing dentin and removing all surface contaminations, including hemostatic agents. Self-etch systems, in contrast, have low acidity (4). It has been demonstrated that if surface contaminants are not removed a proper bond will not be achieved and debonding will occur. In such a case, bacteria, ions and molecules penetrate through the gaps between the tooth structure and restorative materials, resulting in microleakage due to capillarity (7).

Kimmes et al. reported that the use of hemostatic agents ViscoStat Plus (22% ferrous chloride) and ViscoStat (20% ferrous sulfate) has no effect on the shear bond strength to dentin with the use of total-etch adhesive system. The differences in the results of that study and the present one might be attributed to differences in the hemostatic agents, the adhesive resins and the duration of the hemostatic agent application. In that study, ferrous chloride and ferrous sulfate were used for 1 minute and total-etch adhesive was applied (2); however, in the present study, self-etch all-in-one adhesive was used and aluminum chloride hemostatic agent was applied for two minutes.

It is suggested that the effect of various hemostatic agents on the efficacy of self-etch systems with different acidities be evaluated in future studies and composite resin-tooth interface be evaluated under electron microscope.
